# Veno-venous extracorporeal membrane oxygenation (VV ECMO) cannula malposition identified with point-of-care ultrasound

**DOI:** 10.1186/s13089-024-00357-6

**Published:** 2024-05-08

**Authors:** Taylor Becker, Roger D. Struble, Charles Rappaport

**Affiliations:** 1 Department of Internal Medicine, University of Iowa, C123 GH, 200 Hawkins Dr., Iowa City, IA 52242 USA; 2 Department of Internal Medicine, Division of Pulmonary, Critical Care, and Occupational Medicine, University of Iowa, Iowa City, USA

**Keywords:** ECMO, Point-of-care systems, Ultrasound, Cannulation

## Abstract

**Background:**

Point-of-care ultrasound (POCUS) has become a mainstay in the evaluation of critically ill patients in the intensive care unit (ICU). ECMO patients are susceptible to complications during prolonged ICU stay, including cannula malposition, which has deleterious consequences. Although the literature surrounding utility of ultrasound on ECMO patients is expansive, direct comparison between radiographic imaging versus ultrasound for identification of cannula malposition is lacking.

**Case presentation:**

The authors identified four patients with cannula malposition discovered through POCUS that was missed on routine radiographic imaging. Identification and correction of malposition changed their ECMO course.

**Conclusion:**

This case series is the first in literature demonstrating that ultrasound may be superior to radiographic images for ECMO cannula malposition. Further investigation into this subject is warranted.

## Background

Ultrasound has an important role in the management of critically ill patients; however, there are limited guidelines outlining the use in adults requiring extracorporeal membrane oxygenation (ECMO) [1]. Currently, there are no proposed guidelines for regular point-of-care ultrasound (POCUS) evaluations in the adult ECMO patient population resulting in massive institutional variability in its application. Currently, our institution utilizes POCUS daily to identify and even correct possible ECMO complications in a rapid, cost-effective manner. One such complication, cannula malposition, can have tremendous consequences including direct vessel or cardiac trauma [2], recirculation, and liver and splanchnic organ congestion [3, 4] which can result in cerebral hypoperfusion, hemodynamic instability, and even death [5].

To our knowledge, there are no published reports investigating the superiority of ultrasound in the identification of cannula malposition compared to routine radiography in adult ECMO patients. We present the retrospective review in adult ECMO patients where routine radiographic evaluation was reassuring for proper cannula positioning despite frank malposition identified on POCUS. We demonstrate that POCUS may be superior to routine radiography in the monitoring of ECMO cannula position.

## Case presentations

**Patient 1** was a 27-year-old female with Hodgkin’s lymphoma and resulting bleomycin-induced lung injury. She underwent cannulation via dual lumen internal jugular (IJ) for veno-venous (VV) ECMO for progressive and refractory hypoxemia, with line verified intraoperatively. Daily chest radiograph (CXR) commented “ECMO cannula in appropriate position”. After developing increasing evidence of recirculation on her circuit on cannulation day 2, she underwent POCUS evaluation which showed outflow jet positioned distally in the hepatic vein. Recirculation improved with retraction of the cannula under ultrasound guidance, however the patient expired after developing acute right ventricular failure of unclear etiology.

**Patient 2** was a 31-year-old male with rapidly progressive interstitial lung disease secondary to protein alveolar proteinosis. He underwent intubation and VV ECMO cannulation as a bridge to transplant. Two days after dual lumen IJ cannulation, he showed evidence of recirculation and low flows (SpO2 88%, ScVO2 82%, flow 2.1Lpm). CXR commented “dual lumen IJ ECMO cannula with side hole in the right atrium, distal tip in the IVC.” POCUS showed outflow jet positioned in the hepatic vein. Support immediately improved once the cannula was retracted under ultrasound guidance (SpO2 98%, flow 3.1Lpm). Ultimately, patient survived on VV ECMO until lung transplant was performed and is still alive.

**Patient 3** was a 30-year-old-female with acute respiratory distress syndrome (ARDS) secondary to viral pneumonia. Cannulated for VV ECMO via dual lumen IJ. On day 13 of cannulation, had increase in AST/ALT. CXR performed same day reported “ECMO cannulas appear to be in appropriate position.” POCUS showed the return jet had migrated distally and was partially positioned in hepatic vein. He underwent repositioning bedside under ultrasound guidance. The following day, his AST/ALT normalized. She underwent decannulation two days later and survived.

**Patient 4** was a 40-year-old female with ARDS secondary to viral pneumonia, complicated by refractory hypoxemia. She underwent bi-femoral VV ECMO cannulation. On day 8 of ECMO, she began cutting out flow and had marginal support despite fluids. CXR on day 6 and 8 both commented “ECMO cannula terminating in the cavo-atrial junction.” POCUS showed tip of the return cannula distal to the hepatic vein with the return jet partially entering the hepatic vein when it had been in the right atrium on days 1–2 ultrasonographically. Her cannula was exchanged for a dual lumen IJ given hematoma formation around groin sites and remained on ECMO for an additional ten days. On ECMO day 24, she developed progressive acidosis and ECMO support failed, resulting in cardiac arrest and patient demise.

## Discussion

**Fig. 1:**
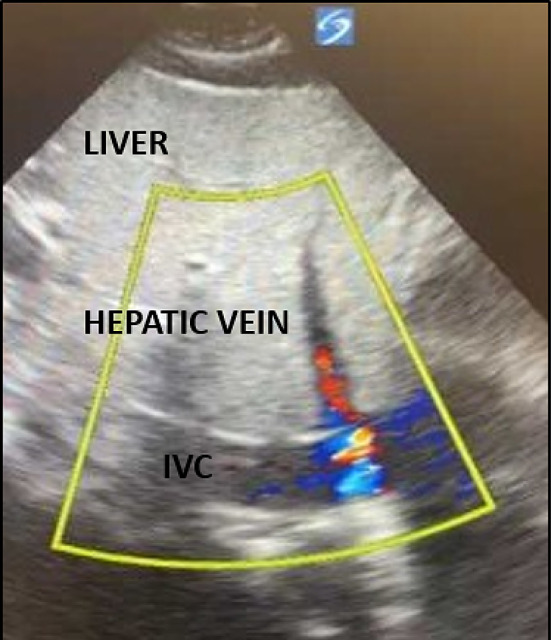
Subcostal IVC view with the return jet positioned in the hepatic vein

**Fig. 2:**
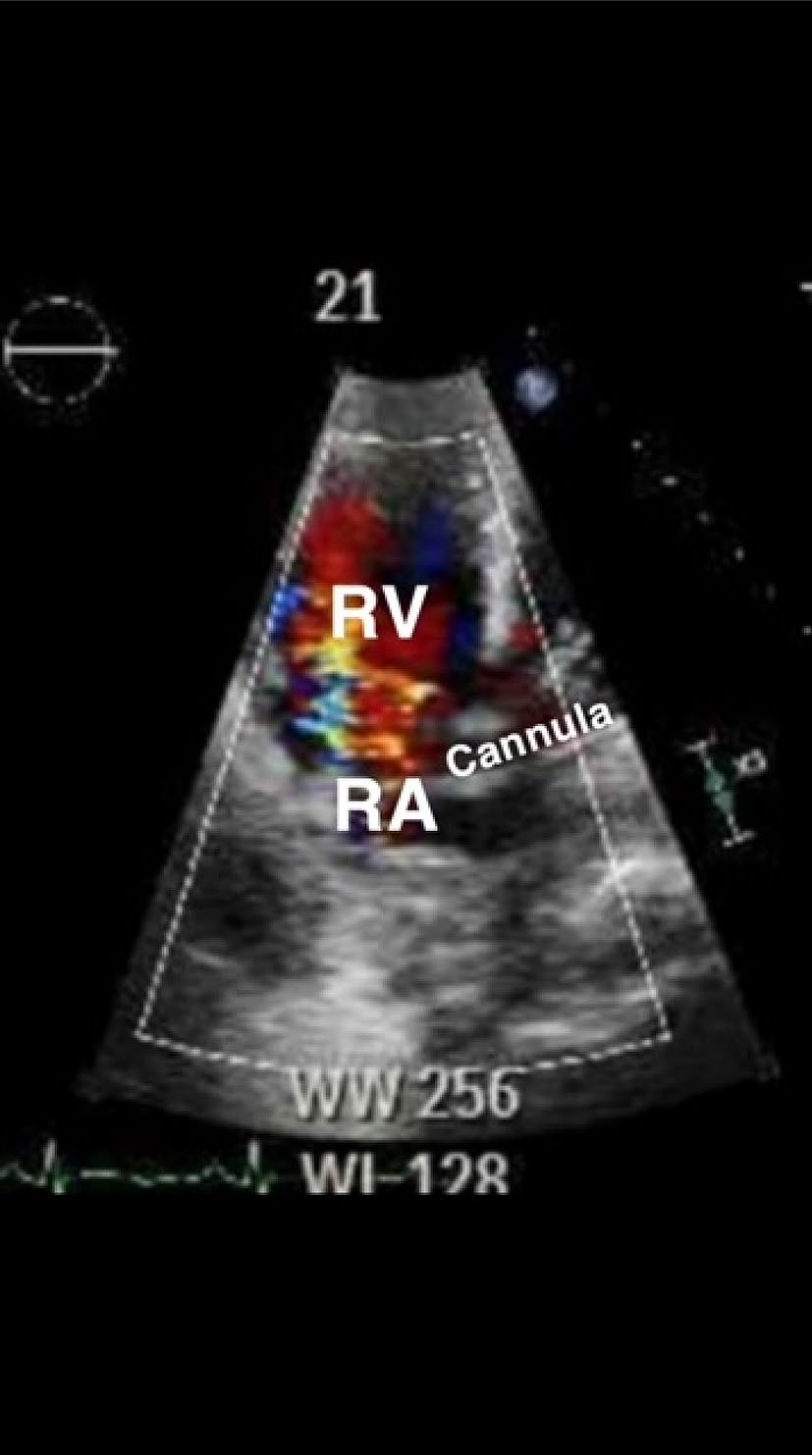
Parasternal right ventricular (RV) inflow view showing the return jet appropriately positioned in the RV

Multiple position papers, including a 2014 paper by the International ECMO Network “recommend selected members of the ECMO team should be trained in vascular and cardiac ultrasonography for insertion, maintenance, and surveillance of the ECMO device.” [6, 7]. Despite this, a vast majority of guidelines from professional societies are either vague or lacking when discussing the optimal imaging modality for monitoring the position of ECMO cannulas [8]. Practices vary institutionally, although frequent radiographic evaluation appears to be commonplace. Despite numerous studies highlighting the value of POCUS in the evaluation and management of critically ill patients [9, 10], prospective studies regarding POCUS in ECMO patients are lacking. To date, there are several case reports and one prospective study that have highlighted the utility of POCUS in the evaluation of ECMO cannula malposition [3, 4, 11, 12] but none have compared it to radiography. Additionally, these studies did not describe malposition identified by POCUS despite a normal radiograph. Our retrospective review suggests that radiographs may miss clinically important malposition that POCUS rapidly identifies. Additionally, POCUS provides several additional advantages when compared to radiography (e.g., color Doppler to view the outflow jet position, ease of access, minimal patient repositioning, live time cannula repositioning). Radiographic evaluation of ECMO cannulas is limited by underlying ARDS, pneumonia, or pulmonary edema-factors that obscure normal mediastinal and bony landmarks [13]. Situational factors, including poor patient positioning or difficulty maneuvering various lines or tubes in the critically ill patient, can lead to changes in the projected cannula tip position (Figs. 1, 2).

In addition to superiority in the evaluation of cannula malposition, ultrasound provides several other benefits. Ultrasound machines are readily available at the bedside in most ICUs, and the use of portable/hand-held ultrasounds is ever-increasing. POCUS is advantageous in facilities where ultrasound or echocardiography is not always available. Ultrasound also reduces unnecessary radiation exposure to patients and staff. It includes real-time imaging before cannulation, during and after cannula repositioning at the bedside, avoiding unstable patient transfers, less expert consultation use, and subsequent economic savings [9]. There is well-defined utility of ultrasound, specifically echocardiography, throughout all stages of ECMO currently defined in literature [5, 14–18].

### Management stage

The use of ultrasound in the management stage of ECMO has the broadest application. In addition to echocardiography, a POCUS exam also includes at least daily assessments of the inferior vena cava (IVC) for volume assessment, cannulated vessels, cannula position(s), surrounding superficial and deep structures, and lungs. Regarding intravascular volume status, providers are tempted to administer additional intravenous fluids (IVF) with sudden decreases in circuit blood flow, mistakenly equating drops in flow to decreased intravascular volume. A plethora of data now indicate a negative fluid balance is associated with improved outcomes in ARDS, a common indication for VV ECMO [19]. Even in the absence of cannula malposition, POCUS can direct a provider to look for other etiologies of decompensation. If POCUS indicates proper cannula position and there is evidence of circuit flow compromise secondary to hypovolemia (negative pressures in the outflow limb, chatter, or collapse of the IVC around the cannula), additional IVF administration could be beneficial [20]. POCUS can also identify clinically relevant cannula site hematomas, peri-cannula thrombus or deep vein thrombosis obstructing circuit flow. Other vascular complications secondary to cannulization including vessel dissection, posterior vessel wall perforation and fistula formation can be identified through POCUS in the hands of an experienced provider [8, 17].

New onset hypotension may be a result of an underlying secondary process for which POCUS is again beneficial. Minor and major hemorrhage is not uncommon in these patients secondary to anticoagulation and platelet dysfunction. Concurrent thrombocytopenia, hepatic dysfunction and renal failure are not uncommon in these critically ill patients, further contributing to coagulopathy [21, 22]. Secondary to prolonged hospital stays, nosocomial infections are common and may contribute to underlying endothelial injury and leakage, further exacerbating hypotension and hypoperfusion. Again, POCUS aids in diagnosis of infections such as pneumonia or parapneumonic effusions [23]. Moreover, pleural ultrasound can assist in the diagnosis of pneumothorax, which is a relatively common cause of decompensation in mechanically ventilated ARDS patients. Other etiologies of shock including new onset cardiac dysfunction, cardiac tamponade, and even pulmonary embolism can be diagnosed with POCUS. Tamponade may occur hours or days after initial cannulation in the setting of anticoagulation initiation, with conventional signs and symptoms not seen secondary to support by the ECMO circuit [14]. Paradoxically, ECMO patients are at high risk of both hemorrhage and thrombosis, with a relatively high rate of PE occurrence estimated at 16.2% [22]. Frequent POCUS evaluation, even in the hemodynamically stable patient, is warranted to ensure that complications such as cannula migration are caught early [5, 20].

### Cannula malposition

In our patients, the first sign of cannula malposition was an abrupt drop in circuit blood flow or new hypoxia. A sudden decrease in flow, typically 1–2 L-per-minute (LPM) from baseline, results in a phenomenon known as ‘suck down’ or ‘chugging.’ [19] POCUS distinguishes between the two most common etiologies of reduced circuit flow: intravascular volume depletion and cannula malposition.

Duration of cannulation for patients on ECMO varies depending on the type of support. VA ECMO runs are shorter than VV ECMO runs; on average, patients on VV ECMO are cannulated for 10–14 days [24]. Accidental movement of cannulas can occur at any time [15]. Cannula malposition is estimated to develop in roughly 5% of ECMO patients and is more common in VV ECMO patients [25]. Patients on VV ECMO commonly undergo physical therapy, stand, walk, and are sometimes subjected to intermittent prone ventilation early in the course of illness. Depending on cannulation configuration, subsequent complications related to malposition have varied consequences.

In the dl Vj-V configuration, minor changes in position can have disastrous consequences. Adequate delivery of oxygenated blood to pulmonary circulation relies on the outflow jet facing the tricuspid valve annulus. Proximal or distal migration of the cannula results in recirculation; a phenomenon in which returned, highly oxygenated blood is immediately drained back into the ECMO circuit. This results in decreased oxygen delivery, global hypoxia, and hypoperfusion [1]. Recirculation can also be seen in single lumen cannula configurations such as Vf-Vj, wherein the femoral drainage cannula migrates superiorly toward the IJ return cannula.

In both dual and single lumen jugular venous cannulas, distal migration of the return jet into the hepatic vein can cause several problems, including hepatic congestion, hepatic vein trauma, or decreased circuit blood flow [3, 4, 26]. One patient in our study had an acute elevation of liver enzymes, prompting a POCUS evaluation that identified distal migration of the dl Vj-V cannula. Re-positioning resulted in hepatic enzyme normalization. Migration or displacement into or through the IVC, right atrium (RA), and right ventricle (RV), have been described [2, 11, 26, 27]. This can occur in any configuration, and can result in low circuit flow, chugging, direct vessel trauma, ventricular rupture, and tamponade.

Our review is not the first to explore the utility of POCUS for cannula malposition in decompensating, seriously ill patients on extracorporeal support. A retrospective observational study performed at Massachusetts General Hospital evaluated 189 medical/cardiac ICU patients who underwent rescue POCUS (r-POCUS) during an episode of clinical decompensation. The most common reason for examination was hypotension (49%), followed by evaluation of cannula position (17%). Cannula malposition was identified in 9 patients (5%) with 8 patients undergoing cannula re-positioning because of the r-POCUS findings [12]. Although this study reinforces the utility of POCUS for evaluation of cannula position, it does not compare ultrasound images with standard radiographic images to determine if malposition was suspected.

Although comparisons between POCUS and radiography in adults on ECMO is lacking, a plethora of research in pediatric ECMO supports ultrasound’s superiority to radiographic evaluation of cannula position. A 2020 retrospective study of 39 infants performed by Pawlowski et al. found that POCUS identified cannula malposition in 19 patients who had an optimally positioned cannula determined by plain radiography (kappa of 0.13); the positive predictive value for plain radiography was only 54% [28]. Malposition occurred in half of the infants evaluated, and 52% of patients with malpositioned cannulas had an intervention after POCUS examination. The discordance between POCUS and plain radiography echoed results from multiple prior studies [29, 30].

### Study limitations

The retrospective nature of this study limits its applications. Additionally, only 4 patients are included, limiting the power of this study. Time between radiograph and POCUS was unable to be determined through chart review, so positional changes could have occurred after radiographs were taken. POCUS examinations are limited by provider training and accuracy. Finally, all 4 patients included were on VV ECMO, which makes this review less applicable to VA ECMO patients. Despite these limitations, we strongly believe that our case series, in addition to the current literature, warrants evaluation of the current dependence on radiographic studies for ECMO cannula malposition.

## Interpretation

This case series indicates that POCUS may be superior to radiography in the identification of ECMO cannula malposition. Additionally, ultrasound remains a fast and readily available tool that provides critical information throughout a patient’s extracorporeal treatment. This includes pre-evaluation and patient selection, cannula insertion, monitoring of progress, detection of complications, determining possibility of recovery, and weaning of support. We recommend a prospective study, directly comparing radiography to ultrasound in ECMO cannula position determination in adults.

## Data Availability

All data generated or analyzed during this study are included in this published article and its supplementary information files.
